# Metabolomics reveals that the cAMP receptor protein regulates nitrogen and peptidoglycan synthesis in *Mycobacterium tuberculosis*†
†Electronic supplementary information (ESI) available. See DOI: 10.1039/d0ra05153e


**DOI:** 10.1039/d0ra05153e

**Published:** 2020-07-10

**Authors:** Yi Liu, Sonia Rebollo-Ramirez, Gerald Larrouy-Maumus

**Affiliations:** a MRC Centre for Molecular Bacteriology and Infection, Department of Life Sciences, Faculty of Natural Sciences, Imperial College London, London, UK. Email: g.larrouy-maumus@imperial.ac.uk

## Abstract

*Mycobacterium tuberculosis* requires extensive sensing and response to environment for its successful survival and pathogenesis, and signalling by cyclic adenosine 3′,5′-monophosphate (cAMP) is an important mechanism. cAMP regulates expression of target genes *via* interaction with downstream proteins, one of which is cAMP receptor protein (CRP), a global transcriptional regulator. Previous genomic works had identified regulon of CRP and investigated transcriptional changes in *crp* deletion mutant, however a link to downstream metabolomic events were lacking, which would help better understand roles of CRP. This work aims at investigating changes at metabolome level in *M. tuberculosis crp* deletion mutant combining untargeted LC-MS analysis and ^13^C isotope tracing analysis. The results were compared with previously published RNA sequencing data. We identified increasing abundances of metabolites related to nitrogen metabolism including ornithine, citrulline and glutamate derivatives, while ^13^C isotope labelling analysis further showed changes in turnover of these metabolites and amino acids, suggesting regulatory roles of CRP in nitrogen metabolism. Upregulation of diaminopimelic acid and its related genes also suggested role of CRP in regulation of peptidoglycan synthesis. This study provides insights on metabolomic aspects of cAMP-CRP regulatory pathway in *M. tuberculosis* and links to previously published transcriptomic data drawing a more complete map.

## Abstract 2

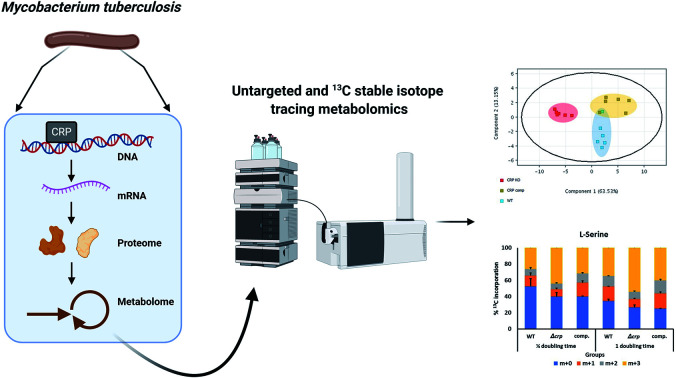
 *Mycobacterium tuberculosis* requires extensive sensing and response to environment for its successful survival and pathogenesis, and signalling by cyclic adenosine 3′,5′-monophosphate (cAMP) is an important mechanism.

## Introduction

*Mycobacterium tuberculosis* (Mtb), the causative agent of tuberculosis (TB), is one of the leading causes of death among infectious diseases, leading to 10 million infections and 1.5 million deaths across the world in 2018.^1^ Latent TB infection is estimated to be found in one quarter of the global population, acting as a potential reservoir for the disease, while emergence of multidrug-resistant TB (MDR-TB) and extensively drug-resistant TB (XDR-TB) resistant to first-line and second-line anti-TB drugs remains a threat to global health. Mtb is transmitted between human *via* inhalation of bacterium-containing droplets, and enter the lungs where it infects pulmonary macrophages.^2^ Its intracellular lifestyle requires extensive sensing of environmental stresses and quick bacterial responses to ensure survival and pathogenicity.^3^ Presence of secondary messengers, such as cyclic nucleotides, guanosine pentaphosphate ((p)pGpp) and nitrogen oxide, allows successful communications between bacterial machineries to achieve this aim.^3^ Cyclic adenosine 3′,5′-monophosphate (cAMP) is a critical signalling molecule first studies in *Escherichia coli* catabolite repression,^4^,^5^ followed by discovery of its wider regulatory roles in bacterial pathogenicity.^6^ Synthesis of cAMP from adenosine triphosphate (ATP) is catalysed by adenylyl cyclases (ACs). The Mtb H37Rv genome encodes 16 ACs, 10 of which have enzymatic activities, enabling integration of multiple stimuli.^7^,^8^ The ability of Mtb to utilize multiple carbon sources reduces its need for catabolite repression,^9^ however the importance of cAMP in Mtb has been shown in both virulence and host interactions.^10^ The high concentration of intracellular cAMP found in Mtb which increases during macrophage infection also suggests its importance.^11^

cAMP regulates expression of target genes *via* interaction with downstream proteins. cAMP-receptor protein (CRP, Rv3676) is one of these proteins identified in Mtb, being activated by direct binding to cAMP and working as a global transcriptional regulator.^3^ It differs from its *E. coli* homolog, in that binding of cAMP to CRP_Mt_ is weaker and does not affect its DNA-binding properties.^12^ Point mutations found in the DNA- and cNMP-binding domains of CRP in *Mycobacterium bovis* BCG inactivate the protein and were linked to lower virulence of the strains.^13^ Deletion of *crp* in Mtb resulted in growth defects in murine macrophages,^14^ while overexpression of *crp* resulted in rapid growth of Mtb under stress conditions.^15^ The putative CRP regulon consisting of 114 genes was predicted using previously-identified *E. coli* CRP binding sites,^16^ and the regulon was extended to 207 potential genes by using promoter sequences from the GlxR regulon, an ortholog of CRP in *C. glutamicum*.^17^ Several genes with important roles in Mtb were found to be regulated by CRP, including *rpfA*, a resuscitation-promoting factor that is involved in resuscitation of bacteria from dormant states,^14^,^18^ and *whiB1*, an essential transcription factor responding to nitrogen oxide.^12^,^19^,^20^ CRP was also found to regulate *serC* pathway in serine metabolism, contributing to slower growth of the mutant which could be restored by supplementation of serine in liquid cultures but not in macrophages.^21^ Kahramanoglou *et al.*, studied the role of CRP on the overall transcriptome by chromatin immunoprecipitation sequencing (ChIP-seq) and RNA sequencing, and identified 191 binding sites in both intragenic and intergenic regions of the Mtb genome. A widespread transcriptional alteration covering more than one fifth of the total genome could by identified in a *crp* mutant.^22^

Previous studies worked on transcriptomic levels of CRP_Mt_ regulation but there has been no downstream metabolomics study showing overall changes in bacterial metabolome in the absence of *crp*.^23^ The aim of this study is to investigate the role of CRP at the metabolomics level. Specifically, untargeted metabolomics was performed to identify changes in the overall metabolome, while ^13^C isotope tracing was performed to measure rate of interconversion of key metabolites. Integration of metabolomics profile with transcriptomic regulation of CRP_Mt_ will help better understand its roles in cAMP signalling, survival and pathogenicity of Mtb.

## Material and methods

### Materials

Unless otherwise stated, all chemicals and reagents were purchased from Sigma-Aldrich.

### Bacterial strains and growth conditions

The *M. tuberculosis* H37Rv parental, knockout for Rv3676 (CRP) and complemented strains were provided by Dr Roger Buxton MRC-National Institute for Medical Research.^22^ Mycobacterial strains were cultured up to mid-exponential phase in 7H9 liquid medium supplemented with 0.5 g l^–1^ fraction V bovine serum albumin, 0.05% tyloxapol, 0.2% dextrose, 0.2% glycerol, and 10 mM NaCl. For metabolomics profiling studies, mycobacteria were cultured on 7H10 agar supplemented with 0.5 g l^–1^ fraction V bovine serum albumin, 0.2% dextrose, 0.2% glycerol and 10 mM NaCl. Throughout the study, mycobacteria were cultured in a shaking incubator set at 125 rpm and 37 °C.

### Metabolite extraction experiments

For targeted and untargeted metabolomic profiling studies, mycobacteria were cultured as described.^24^–^26^ Briefly, mycobacteria were initially grown in 7H9 liquid medium containing the carbon sources of interest until the OD_600_ reached ∼0.8–1. Bacteria were then inoculated onto 0.22 μm nitrocellulose filters under vacuum filtration. Mycobacterial-laden filters were then placed on top of chemically equivalent agar media (described above) and allowed to grow at 37 °C for 5 doubling times to generate enough biomass for targeted metabolomics studies. Filters were then transferred into 7H10 plates supplemented with 0.5 g l^–1^ fraction V bovine serum albumin, 0.2% dextrose and 0.2% glycerol, 10 mM NaCl. Bacteria were metabolically quenched by plunging the filters into the extraction solution composed of acetonitrile/methanol/H_2_O (2 : 2 : 1) pre-cooled to 4 °C. Small molecules were extracted by mechanical lysis of the entire bacteria-containing solution with 0.1 mm acid-washed zirconia beads for 1 min using a FastPrep (MPBio®) set at 6.0 m s^–1^. Lysates were filtered twice through 0.22 μm Spin-X column filters (Costar®). Bacterial biomass of individual samples was determined by measuring the residual protein content of the metabolite extracts using the BCA assay kit (Thermo®).^9^,^27^

### Liquid-chromatography-mass spectrometry

Aqueous normal phase liquid chromatography was performed using an Agilent 1290 Infinity II LC system equipped with a binary pump, temperature-controlled auto-sampler (set at 4 °C) and temperature-controlled column compartment (set at 25 °C) containing a Cogent Diamond Hydride Type C silica column (150 mm × 2.1 mm; dead volume 315 μl). A flow rate of 0.4 ml min^–1^ was used. Elution of polar metabolites was carried out using solvent A consisting of deionized water (resistivity ∼18 MΩ cm) and 0.2% acetic acid and solvent B consisting of 0.2% acetic acid in acetonitrile. The following gradient was used: 0 min 85% B; 0–2 min 85% B; 3–5 min to 80% B; 6–7 min 75% B; 8–9 min 70% B; 10–11 min 50% B; 11.1–14 min 20% B; 14.1–25 min hold 20% B followed by a 5 min re-equilibration period at 85% B at a flow rate of 0.4 ml min^–1^. Accurate mass spectrometry was carried out using an Agilent Accurate Mass 6545 QTOF apparatus. Dynamic mass axis calibration was achieved by continuous infusion, post-chromatography, of a reference mass solution using an isocratic pump connected to an ESI ionization source operated in the positive and negative-ion mode. The nozzle voltage and fragmentor voltage were set at 2000 V and 100 V, respectively. The nebulizer pressure was set at 50 psig, and the nitrogen drying gas flow rate was set at 5 l min^–1^. The drying gas temperature was maintained at 300 °C. The MS acquisition rate was 1.5 spectra per sec, and *m*/*z* data ranging from 50–1200 were stored. This instrument enabled accurate mass spectral measurements with an error of less than 5 parts-per-million (ppm), mass resolution ranging from 10 000–45 000 over the *m*/*z* range of 121–955 atomic mass units, and a 100 000-fold dynamic range with picomolar sensitivity. The data were collected in the centroid 4 GHz (extended dynamic range) mode, for optimal data storage and improved the speed of isotologues extractions. Detected *m*/*z* were deemed to be identified metabolites based on unique accurate mass-retention time and MS/MS fragmentation identifiers for masses exhibiting the expected distribution of accompanying isotopomers. Typical variation in abundance for most of the metabolites remained between 5 and 10% under these experimental conditions.

### ^13^C-Labelling analysis

Under the experimental conditions described above using [U-^13^C_3_] glycerol (99%) and [U-^13^C_6_] glucose (99%), the extent of ^13^C labelling for each metabolite was determined by dividing the summed peak height ion intensities of all ^13^C-labelled species by the ion intensity of both labelled and unlabelled species using the software Agilent Profinder version B.8.0.00 service pack 3.

### Statistical analysis

Data are presented as the mean ± standard error of the mean from 2 biological replicates and 3 technical replicates per condition. Unpaired two-tailed Student's *t*-tests were used to compare values, with *p* < 0.05 considered significant.

### Biological safety considerations

Bacteria were handled within a Class-I safety-level cabinet equipped with HEPA filters.

## Results and discussions

### Untargeted metabolomics revealed minimal changes in overall metabolome in *M. tuberculosis* Δ*crp*

**Fig. 1:**
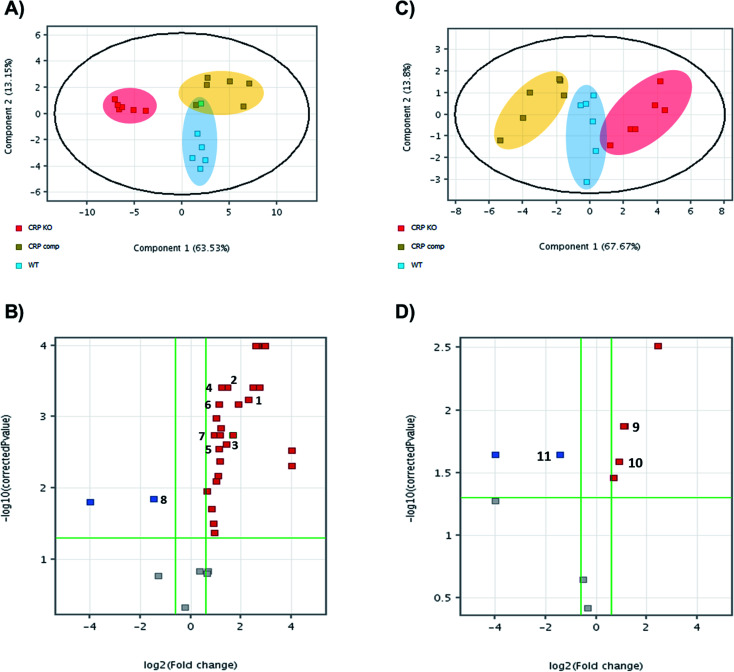
Untargeted metabolomics reveals that CRP knockout leads to metabolome remodelling. Principal component analysis (PCA) plots of untargeted metabolomics analysis in (A) positive mode and (C) negative mode. Each dot on plots indicated individual sample and was coloured and grouped by conditions. WT is displayed in blue, knockout in red and complemented in yellow. Volcano plots of untargeted metabolomics analysis (B) positive mode and (D) negative mode. Each dot indicates a metabolite, with red colour indicating significant upregulation compared with WT, blue for downregulation and grey for no significant differences between groups. Numbers indicate annotated metabolites as listed in Table 1.

Bacteria, wild-type, knock-out and complemented strains, were harvested and metabolites extracted for untargeted metabolomics by LC-MS. Features of metabolites were extracted by MassHunter Profinder 8.0 and were analysed and compared between groups in Mass Profinder Professional 12.0 (Agilent technologies). Principal component analysis (PCA) plots exhibit separation of the *M. tuberculosis* Δ*crp* group from wild-type and complemented in both positive and negative modes (Fig. 1A and C). In positive in mode, the *M. tuberculosis* Δ*crp* samples showed clear separation with WT and complemented groups, which were partially overlapped. The differences between groups were less significant in negative mode. Volcano plots were also generated in both positive and negative ion modes to visualise changes in metabolite abundances in either *M. tuberculosis* Δ*crp* or complemented groups compared with WT. *M. tuberculosis* Δ*crp* showed a general upregulation greater than 1.5-fold in positive mode and *p* < 0.05, while only 2 metabolites were found downregulated. There were less metabolites passing cut-offs of 1.5-fold change and *p* < 0.05 in negative mode. When comparing complemented group and WT, only two metabolites passed both cut-offs in negative mode while others failed due to insignificance (*p* > 0.05), suggesting close similarity between metabolomes of complemented and WT bacteria as expected (ESI Table 1†).

The significant *m*/*z* values were then searched in ECMDB (; https://ecmdb.ca) and METLIN (; https://metlin.scripps.edu) for annotation. Those metabolites were also searched in KEGG pathway database (; https://www.genome.jp/kegg/pathway.html) to ensure existence of relative pathways in Mtb metabolome. A total of 9 metabolites in positive mode and 3 in negative mode were annotated that showed significant changes (absolute FC > 1.5 and *p* < 0.05) in abundances in mutants compared with the parental strain (Table 1). Identities of these metabolites were confirmed by MS/MS analysis (data not shown). As shown in volcano plots, those metabolites showing significant FC in *M. tuberculosis* Δ*crp* group did not show significant differences in abundance between complemented group and WT, suggesting successful complementation of mutant. Then, the metabolomics data generated was correlated to transcriptomic data previously published by Kahramanoglou *et al.*, 2014, by searching for metabolite-related genes in mycobrowser (; https://mycobrowser.epfl.ch/) and referring back to RNA-sequencing data.^22^

**Table 1:** List of metabolites annotated in untargeted analysis. Identities of compounds were annotated using ECMDB (http://ecmdb.ca/) and METLIN (; https://metlin.scripps.edu/) online databases, and were further confirmed by MS/MS. Differences between experimental and actual *m*/*z* values were shown as Δppm

Compound number	Compound name	*m*/*z*	Adduct	Δppm	log_2_ FC ([Δcrp] *vs.* [WT])	*p* (corr)	log_2_ FC ([comp.] *vs.* [WT])	*p* (corr)	Retention time (min)
1	*N*-Acetyl-l-glutamate 5-semialdehyde	174.0761	[M + H]^+^	0	10.79965	4.87 × 10^–3^	9.342301	5.56 × 10^–2^	10.48
2	*N*-Acetyllactosamine	384.1500	[M + H]^+^	0	2.263255	5.78 × 10^–4^	–0.37745	7.78 × 10^–1^	5.66
3	Diaminopimelic acid	191.1026	[M + H]^+^	0	1.435697	3.90 × 10^–4^	–0.06216	9.53 × 10^–1^	10.48
4	Nicotinate d-ribonucleoside	256.0816	[M + H]^+^	0	1.400408	2.45 × 10^–3^	–0.41801	7.40 × 10^–1^	8.99
5	N_2_-Succinyl-*l*-ornithine	233.1132	[M + H]^+^	0	1.220208	3.90 × 10^–4^	–0.37384	7.46 × 10^–1^	9.41
6	N1-Methyladenine	150.0774	[M + H]^+^	3	1.108941	2.81 × 10^–3^	0.287358	7.46 × 10^–1^	10.54
7	4-Methylene-l-glutamine	159.0765	[M + H]^+^	0	1.107723	6.76 × 10^–4^	–0.44266	7.40 × 10^–1^	10.99
8	Citrulline	176.1030	[M + H]^+^	0	0.916007	1.79 × 10^–3^	–0.49898	7.40 × 10^–1^	10.95
9	*N*-Acetylleucine	174.1125	[M + H]^+^	0	–1.47388	1.43 × 10^–2^	0.497911	7.40 × 10^–1^	1.23
10	Malate	133.0142	[M + H]^–^	0	1.117916	1.33 × 10^–2^	–0.8483	1.08 × 10^–1^	1.14
11	Ornithine	131.0827	[M + H]^–^	2	0.882035	2.59 × 10^–2^	–0.50823	2.12 × 10^–1^	10.94
12	2,3-Dihydro-2,3-dihydroxybenzoic acid	155.0350	[M + H]^–^	0	–1.44515	2.26 × 10^–2^	0.762619	2.14 × 10^–1^	8.25

**Fig. 2:**
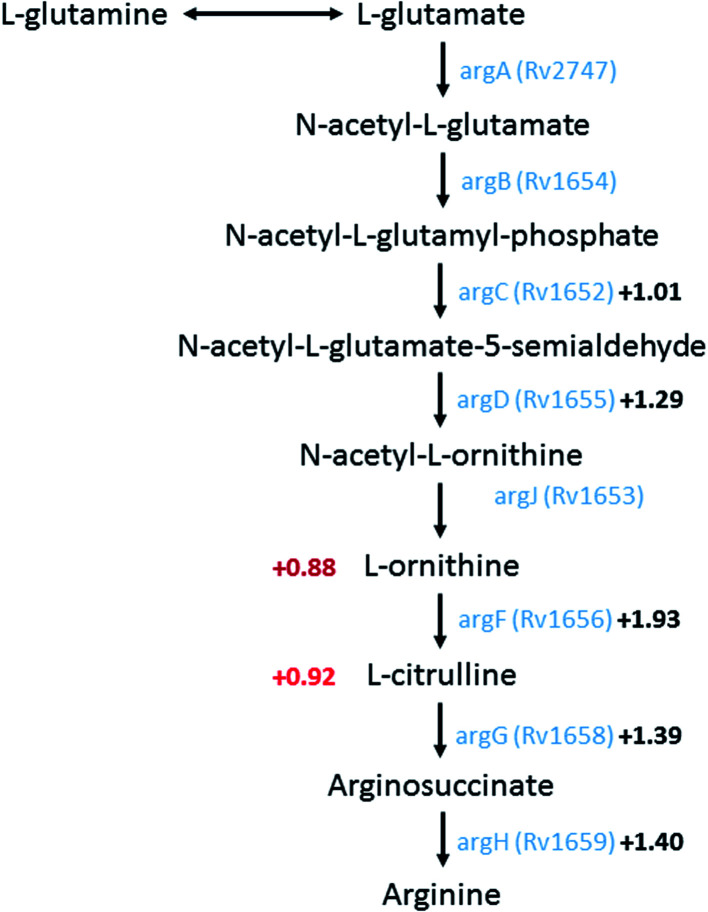
A schematic metabolic pathway showing arginine biosynthesis in *M. tuberculosis*. Logarithmic fold changes (log_2_ FC) of metabolites that were identified in untargeted metabolomics are shown on the left in red. Genes encoding enzymes of the corresponding reaction were shown in blue with the log_2_ FC in expression levels shown in black according to Kahramanoglou and colleagues.^22^ Pathway data acquired from KEGG pathway (; https://www.genome.jp/kegg/pathway.html) and Mycobrowser (; https://mycobrowser.epfl.ch/).

A group of metabolites related to nitrogen metabolism could be annotated showing increased abundance in the *M. tuberculosis* Δ*crp* mutant. l-Ornithine (*m*/*z* 131.0827) was upregulated 0.88 log_2_ fold (log_2_ FC), and citrulline (*m*/*z* 176.103) upregulated 0.92 log_2_ fold. The two metabolites are part of the urea cycle related to synthesis of arginine and recycling of nitrogen. Previous RNA sequencing data suggested an overall upregulation of arginine biosynthesis involving *argBCDFGHJ* genes, with all of them except *argB* showing significantly upregulated expression levels. Upregulation (log_2_ FC = 1.93) of *rv1656* (*argF*), a probable ornithine carbamoyltransferase converting ornithine to citrulline, may be related to upregulation in citrulline abundance. Meanwhile, subsequent production of arginosuccinate from citrulline by arginosuccinate synthase *argG* (*rv1658*) is also upregulated with the corresponding gene showing significant upregulation (log_2_ FC = 1.39). *N*_2_-Succinyl-*l*-ornithine (*m*/*z* 233.1132) was the breakdown intermediate of arginine to form back to glutamate, and showed upregulation of 1.22 log_2_ fold in the knockout mutant. In conclusion, higher abundances of metabolites related to nitrogen metabolism and arginine synthesis/breakdown are found in *M. tuberculosis* Δ*crp* mutant and correlated to higher expression levels of related enzymes, especially those closely located on the Mtb genome, confirming regulation by CRP as a transcriptional regulator (Fig. 2).

Diaminopimelic acid (DAP, *m*/*z* 191.1026) is the building block of mycobacterial cell wall, linking layers of *N*-acetylglucosamine (GlcNAc) and *N*-acetylmuramic acid (MurNAc).^28^ It was found upregulated by 1.44 log_2_ fold in the *M. tuberculosis* Δ*crp* mutant. Among enzymes involved in synthesis of DAP, lysine and peptidoglycan, the tetrahydrodipicolinate *N*-succinyltransferase DapD (Rv1201c) and the probable succinyl-diaminopimelate desuccinylase *dapE* (Rv1202), the two enzymes responsible for upstream synthesis of DAP, showed mild upregulations (log_2_ FC = 0.67 and 0.62, respectively) in *M. tuberculosis* Δ*crp*, while the other enzymes in the pathway did not show significant changes in expression. Genes of other members of the pathway (*dapA*/*B*/*C*/*F*) are located far from loci of *dapD*/*E* and was not regulated by binding of CRP.

Malate (*m*/*z* 133.0142) was the only metabolite in the TCA cycle identified by untargeted metabolomics, with abundance upregulated by 1.18 log_2_ fold. Among genes related to metabolism around malate, the malate synthase G *glcB* (*rv1837c*) and a probable malate:quinone oxidoreductase *mqo* (*rv2852c*) responsible for conversion of malate to glyoxylate and oxaloacetate respectively, showed downregulations in expression (log_2_ FC = –0.66 and –0.87, respectively), however changes in abundances of corresponding products were not observed in untargeted metabolomics.

In conclusion, changes in the *M. tuberculosis* Δ*crp* metabolome were minor and could be correlated to minor changes in the transcriptome as reported.^22^ Our untargeted metabolomic data showed higher abundances of metabolites related to nitrogen metabolism, which could be correlated to higher expression levels of mRNA of enzymes in the pathways. Higher abundances of diaminopimelic acid also implicated CRP in the regulation of peptidoglycan synthesis. On the other hand, although changes in genes involved in central carbon metabolism were found in the genomic studies, there were only limited changes seen at the metabolomics level.

Although untargeted metabolomics provides the abundance of different metabolites within metabolic pathways, several metabolic changes do not *a fortiori* result in an increase or a decrease in the metabolite level, as seen by our previous data. Indeed, stable isotope tracing (*e.g.* ^13^C) provides information not revealed using conventional untargeted metabolomics by measuring the rates of metabolite interconversion as a readout of metabolic enzyme regulations. This makes stable isotope tracer studies a powerful option to probe metabolic changes in the *M. tuberculosis* Δ*crp* mutant.

### ^13^C isotope labelling experiments reveal an increase in turn-over of nitrogen-metabolism metabolites in *M. tuberculosis* Δ*crp*

**Fig. 3:**
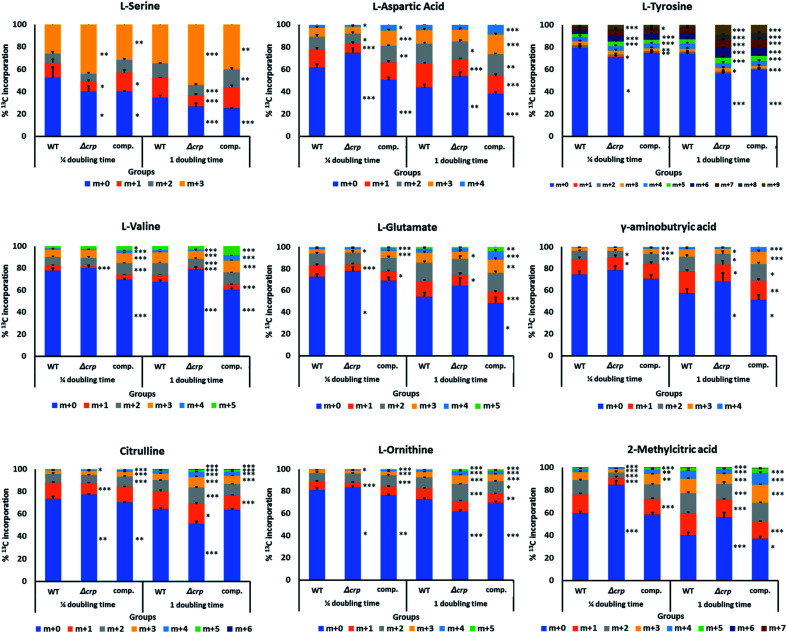
Metabolites identified in ^13^C labelling analysis with significant changes in carbon incorporation between *crp* mutant and WT. Percentage incorporation for each condition was averaged from 2 biological and 3 technical replicates and standard deviations are shown as upward error bars on each fraction of ^13^C labelling. Two-tailed *t*-tests were performed between samples of Δcrp/comp. with WT in different time points, and levels of significance for each labelled fraction were expressed as asterisks on the right of corresponding bar (*: *p* < 0.05; **: *p* < 0.01; ***: *p* < 0.001).

[U-^13^C_3_] glycerol and [U-^13^C_6_] glucose were used as carbon sources to be incorporated into central carbon metabolism through glycolysis and the TCA cycle, and later into amino acids and downstream metabolites. Samples were taken at a 1/4 and 1 doubling time after ^13^C supplementation in each group. An in-house compound database and library containing major metabolites in the TCA cycle and nitrogen metabolism was used to extract the isotopologue profiles of the metabolites of interest (Fig. 3 and ESI Fig. 2†).

Among metabolites investigated, malate and 2-methylcitrate were found with significant changes in ^13^C label incorporation between *M. tuberculosis* Δ*crp* and parental groups with a 10–20% reduction. The lower rate of incorporation into malate, together with its higher abundances as found in untargeted metabolomics, could be explained by reduced activities of malate-converting enzymes, such as Rv1837c and Rv2852c, as discussed earlier. 2-Methylcitrate is a metabolite involved in metabolism of propionate and could be synthesised from propanoyl-CoA and oxaloacetate *via* methylcitrate synthase PrpC (Rv1131). However, the expression level of *prpC* was upregulated by more than 4-fold (log_2_ FC = 2.01), which does not explain the reduction of carbon turnover in the metabolite. The previous transcriptomic analysis also suggested changes in expression levels of enzymes related to glycolysis, pentose phosphate pathway and TCA cycle,^22^ however in our metabolomics study, the changes in either abundances or turnover of related metabolites were minimal. A possible explanation is that the ability of Mtb to respond to and co-catabolise multiple carbon sources led to changes in different aspects of carbon metabolism, and differences in growth conditions could enhance such differences.

Previous untargeted metabolomics suggested upregulation of nitrogen metabolism. We therefore investigated incorporation of carbon into related metabolites. Glutamine is the major nitrogen donor in Mtb and can be converted to glutamate *via* glutamate synthase (GltB).^29^ In our labelling assay glutamine did not show changes in turnover. After ¼ doubling time incubation with ^13^C-labelled carbon sources, 27% of glutamate was labelled in at least one carbon atom, and the number increased to 46% after 1 doubling time. Similar proportions of incorporation were achieved in complemented bacteria, while *M. tuberculosis* Δ*crp* showed a lower rate of incorporation, reaching 36% after 24 hours. The isotopologue profile also indicated lower proportion of labelling in m + 4 fraction, suggesting lower turnover rate of the metabolite in *M. tuberculosis* Δ*crp*. ^13^C incorporation into gamma-aminobutyric acid (GABA), a metabolite closely linked to glutamate and involve in interconversion between glutamate and TCA cycle metabolites *via* the GABA shunt, was also decreased by 26% in *M. tuberculosis* Δ*crp* with a lower percentage of labelling in m + 3 and m + 4 forms. Opposite changes in incorporation was found in urea cycle metabolites citrulline and ornithine, with the mutant showing an approximately 40% increase in overall incorporation after 1 doubling time. Higher proportions of labelling in m + 2 and m + 3 fractions could be found in both metabolites, indicating higher rates of turnover. The result might be correlated with untargeted metabolomics, with increased urea cycle activity leading to higher abundances and turnover rates of ornithine and citrulline. Meanwhile, lower turnover of glutamate and its related metabolites suggested reduced activity of related pathways, while the glutamine pool of might be used for maintaining nitrogen availability though glutamate.

A group of amino acids including tyrosine, valine, alanine and aspartic acid were found to have changes in levels of ^13^C incorporation and isotopologue distribution, while most of them showed lower turnover in *M. tuberculosis* Δ*crp* compared with WT, the levels of incorporation were similar between complemented and WT bacteria, confirming the complementation of our phenotype. This could be explained as the result of abnormal function of nitrogen metabolism and slower growth of *M. tuberculosis* Δ*crp.*^14^ Serine was found to have similar overall levels of labelled metabolites among groups, however when comparing fractions of differently-labelled molecules, the higher proportion of fully-labelled serine (m + 3) could be found in *M. tuberculosis* Δ*crp* (50%) compared with WT or complemented bacteria (30%), suggesting a higher rate of turnover. Serine provides the backbone for synthesis of other amino acids including glycine, cysteine and tryptophan.^21^,^30^ It is also the direct indicator of glycolysis as it is related to the pyruvate pool in central carbon metabolism^29^ and the serine dehydratase gene *sdaA* (Rv0069) converting serine to pyruvate was upregulated (log_2_ FC = 2.57).^22^ A higher turnover of serine was therefore related to maintenance of carbon metabolism and amino acid synthesis levels in *M. tuberculosis* Δ*crp*. Serine was also predicted to be an important nitrogen and carbon source using *in silico* approaches, with multiple metabolic pathways converging in *Mycobacterium* species.^32^ SerC (Rv0884) is a phosphoserine aminotransferase responsible for interconversion from glutamate and 3-phospho-hydroxypyruvate to phosphoserine and 2-oxoglutarate, and was identified as an essential gene in Mtb.^31^ Phosphoserine is then used for production of serine through phosphoserine phosphatase SerB (Rv3042c). Previous studies investigated the regulatory effect of CRP on the *serC* pathway in Mtb and found that the mutant showed decreased expression of *serC* and its divergently transcribed gene Rv0885 with impaired bacterial growth which could be complemented by supplementation with serine, glycine or cysteine.^21^,^22^ Here our result suggested that although *serC* pathway is reduced, the abundance of intracellular serine remained stable, perhaps supplemented from other routes of serine metabolism, further reinforcing the importance of this amino acid in *M. tuberculosis*.^29^,^32^

## Conclusions

Overall, our study analysed the metabolome of the *M. tuberculosis* Δ*crp* mutant in terms of metabolite abundance and turnover, and established correlations with previous transcriptomic results. Knockout of *crp* in Mtb affected nitrogen metabolism as shown by the higher abundances and differential ^13^C turnover rates of related metabolites, while central carbon metabolism was minimally affected. Moreover, changes in the abundance of diaminopimelic acid and upregulation of related genes suggested possible remodelling of peptidoglycan.^32^

In order to validate the changes observed in the present study, one could use assay to increase the intracellular cAMP levels which CRP will respond to and determine if the pathways mentioned here are affected by the changes in cAMP levels. Such approach can be achieved by the stimulation of Mtb by adenylate cyclases activator such as Forskolin which has been shown to stimulate the adenylated cyclase Rv1625c (ref. 33 and 34) or the cAMP analogue, dibutyryl-cAMP, analogue which is less hydrophobic than cAMP and is therefore able to pass through cell membranes, where it accumulates intracellularly as mono-butyryl-cAMP.^35^–^37^ Further, as one of the pathway is the synthesis of the precursor of peptidoglycan, one could investigate the antimicrobial susceptibility of *M. tuberculosis* Δc*rp* to antimicrobials such as d-cycloserine which targets alanine racemase and d-alanine ligase^38^ and vancomycin which binds the d-Ala-d-Ala terminal of the growing peptide chain.^39^ Based on the data presented here, we can anticipate an increase in susceptibility of *M. tuberculosis* Δc*rp* compared to the parental strain.

Although some of these metabolome results could be mapped to changing expression levels of enzymes, this was not always the case, and enzyme abundance and post-translational modifications should be also taken into consideration. Therefore, to link the phenotypes observed *in vivo*, further studies using different carbon sources such as fatty acids are needed, while system biology approaches to probe variation between the metabolic states of different strains or different growing conditions could also be utilised.

## Author contribution

YI, SRR and GLM conceived, designed experiments, performed experiments, analysed data, and wrote the paper.

## Funding

This work was supported by an EPSRC-EMBRACE pump-priming award (EP/M027007/1) and by the ISSF Wellcome Trust (105603/Z/14/Z). Sonia Rebollo Ramirez was funded by the Department of Life Sciences from the Faculty of Natural Sciences Imperial College London, UK.

## Conflicts of interest

The authors declare that they have no conflicts of interest regarding the content of this article.

## Supplementary Material

Supplementary information

Supplementary information
