# Supplementary material for: Metabolomics reveals that the cAMP receptor protein regulates nitrogen and peptidoglycan synthesis in Mycobacterium tuberculosis
Source: RSC Adv. 2020 Jul 10;10(44):26212–9. doi: 10.1039/d0ra05153e (PMC7938724; doi:10.1039/d0ra05153e)
Supplement: RA-010-D0RA05153E-s001 [file RA-010-D0RA05153E-s001.pdf]

Electronic Supplementary Material (ESI) for RSC Advances.
This journal is © The Royal Society of Chemistry 2020

Supplementary Tabel 1: List of features identified from untargeted metabolomics

Mass	Adduct	Log_2FC ([Δcrp] vs [WT])	p (corr)	Log_2FC ([comp.] vs [WT])	p (corr)	Retention time (min)
243.2567	$[M+H]^+$	11.07302	2.96×10^{-3}	9.297014	7.40×10^{-1}	11.72
173.0687	$[M+H]^+$	10.79965	4.87×10^{-3}	9.342301	7.40×10^{-1}	10.48
824.6176	$[M+H]^+$	2.922421	5.13×10^{-5}	0.557369	7.40×10^{-1}	1.04
341.133	$[M+H]^+$	2.723432	3.90×10^{-4}	-0.50562	7.47×10^{-1}	13.06
264.0781	$[M+H]^+$	2.663202	6.29×10^{-6}	0.406375	7.40×10^{-1}	8.37
788.5954	$[M+H]^+$	2.583859	5.13×10^{-5}	0.424467	7.40×10^{-1}	0.95
806.6061	$[M+H]^+$	2.480362	3.90×10^{-4}	0.297174	8.80×10^{-1}	0.98
383.143	$[M+H]^+$	2.263255	5.78×10^{-4}	-0.37745	7.78×10^{-1}	5.66
298.2049	$[M+H]^+$	1.869965	6.76×10^{-4}	0.733005	7.40×10^{-1}	0.99
635.3895	$[M+H]^+$	1.652907	1.79×10^{-3}	-0.2927	8.28×10^{-1}	8.29
190.0955	$[M+H]^+$	1.435697	3.90×10^{-4}	-0.06216	9.53×10^{-1}	10.48
255.0744	$[M+H]^+$	1.400408	2.45×10^{-3}	-0.41801	7.40×10^{-1}	8.99
232.1061	$[M+H]^+$	1.220208	3.90×10^{-4}	-0.37384	7.46×10^{-1}	9.41
310.0834	$[M+H]^+$	1.195456	1.44×10^{-3}	-0.29223	7.52×10^{-1}	8.61
443.1292	$[M+H]^+$	1.146864	4.16×10^{-3}	-0.46805	7.40×10^{-1}	4.13
114.0788	$[M+H]^+$	1.141327	1.79×10^{-3}	-0.01182	9.95×10^{-1}	10.99
149.0696	$[M+H]^+$	1.108941	2.81×10^{-3}	0.287358	7.46×10^{-1}	10.54
158.0691	$[M+H]^+$	1.107723	6.76×10^{-4}	-0.44266	7.40×10^{-1}	10.99
110.1096	$[M+H]^+$	1.06021	6.81×10^{-3}	-0.66368	7.40×10^{-1}	1.24
112.0637	$[M+H]^+$	1.000433	1.05×10^{-3}	-0.42727	7.40×10^{-1}	10.99
128.1201	$[M+H]^+$	0.987063	7.97×10^{-3}	-0.71347	7.40×10^{-1}	1.23
108.0938	$[M+H]^+$	0.935972	4.27×10^{-2}	-0.91592	7.40×10^{-1}	1.13
175.0958	$[M+H]^+$	0.916007	1.79×10^{-3}	-0.49898	7.40×10^{-1}	10.99
239.0561	$[M+H]^+$	0.887432	3.15×10^{-2}	-9.28473	7.40×10^{-1}	1.21
550.3481	$[M+H]^+$	0.815958	1.94×10^{-2}	-0.57422	7.40×10^{-1}	11.01
669.1888	$[M+H]^+$	0.663414	1.49×10^{-1}	-1.86174	7.40×10^{-1}	9.34
669.1878	$[M+H]^+$	0.639118	1.58×10^{-1}	-1.83435	7.40×10^{-1}	9.34
290.1476	$[M+H]^+$	0.628497	1.11×10^{-2}	-0.53465	7.40×10^{-1}	11.08
197.1165	$[M+H]^+$	0.34548	1.49×10^{-1}	-1.07934	7.40×10^{-1}	15.05
125.0838	$[M+H]^+$	-0.23284	4.66×10^{-1}	1.194887	7.40×10^{-1}	11.141
229.1191	$[M+H]^+$	-1.28964	1.69×10^{-1}	2.513863	7.40×10^{-1}	8.07
173.1053	$[M+H]^+$	-1.47388	1.43×10^{-2}	0.497911	7.40×10^{-1}	1.23
233.0121	$[M+H]^+$	-9.04422	1.58×10^{-2}	2.305093	7.40×10^{-1}	8.22
401.1522	$[M-H]^-$	2.413918	3.03×10^{-3}	-0.69845	2.14×10^{-1}	12.99
134.0214	$[M-H]^-$	1.117916	1.33×10^{-2}	-0.8483	1.08×10^{-1}	1.141
190.0949	$[M-H]^-$	1.093205	1.33×10^{-2}	-0.18443	5.76×10^{-1}	10.38
175.0954	[M-H]-	0.88486	2.59×10^{-2}	-0.68316	1.21×10^{-1}	10.94
132.0896	$[M-H]^-$	0.882035	2.59×10^{-2}	-0.50823	2.12×10^{-1}	10.95
349.0603	$[M-H]^-$	0.691737	3.47×10^{-2}	-0.73417	1.21×10^{-1}	10.93
514.1291	$[M-H]^-$	-0.35055	3.79×10^{-1}	-2.14104	4.50×10^{-3}	4.12
514.1294	$[M-H]^-$	-0.51712	2.25×10^{-1}	-2.26139	4.50×10^{-3}	3.84
155.0344	[M-H]-	-1.44515	2.26×10^{-2}	0.762619	2.14×10^{-1}	8.25
166.0473	$[M-H]^-$	-7.36134	5.31×10^{-2}	7.413596	1.08×10^{-1}	1.78
406.9767	$[M-H]^-$	-8.93151	2.26×10^{-2}	4.587288	2.12×10^{-1}	8.29

Supplementary Table 2: Personal Compound Database Library used in stable isotope tracing analysis.

Compound Name	Chemical Formula	Experimental m/z (neg)	Δppm	Retention Time (min)	METLIN	KEGG
Glycine	$C_2H_5NO_2$	74.0248	0.0	9.70	20	C00037
Pyruvic acid	$C_3H_4O_3$	87.0093	-5.7	1.03	117	C00022
L-Alanine	$C_3H_7NO_2$	88.0404	0.0	10.74	11	C00041
(R)-Lactate	$C_3H_6O_3$	89.0244	0.0	1.12	63226	C00256
γ-Aminobutryic acid	$C_4H_9NO_2$	102.0561	0.0	9.17	279	C00334
L-Serine	$C_3H_7NO_3$	104.0354	-1.0	9.75	30	C00065
L-Proline	$C_5H_9NO_2$	114.0561	0.0	9.62	29	C00148
Fumaric acid	$C_4H_4O_4$	115.0037	0.0	1.13	3242	C00122
L-Valine	$C_5H_{11}NO_2$	116.0717	0.0	10.48	35	C00183
Succinic acid	$C_4H_6O_4$	117.0195	-1.7	1.08	114	C00042
L-Threonine	$C_4H_9NO_3$	118.051	0.0	9.63	32	C00188
Itaconic acid	$C_5H_6O_4$	129.0193	0.0	0.78	44764	C00490
L-Isoleucine	$C_6H_{13}NO_2$	130.0874	0.0	10.48	23	C00407
L-Leucine	$C_6H_{13}NO_2$	130.0874	0.0	10.09	24	C00123
L-Ornithine	$C_5H_{12}N_2O_2$	131.0827	-0.8	10.97	45121	C00077
L-Aspartic Acid	$C_4H_7NO_4$	132.0303	-0.8	8.06	15	C00049
Malic acid	$C_4H_6O_5$	133.0144	-1.5	1.14	118	C03668
Adenine	$C_5H_5N_5$	134.0473	-0.7	11.40	85	C00147
Oxoglutaric acid	$C_5H_6O_5$	145.0144	-1.4	1.01	119	C00026
Glutamine	$C_5H_{10}N_2O_3$	145.0621	-1.4	10.26	18	C00064
L-Lysine	$C_6H_{14}N_2O_2$	145.0982	0.7	13.89	25	C00047
L-Glutamate	$C_5H_9NO_4$	146.0463	-2.7	9.19	19	C00025
Guanine	$C_5H_5N_5O$	150.042	0.7	7.22	315	C00242
L-Histidine	$C_6H_9N_3O_2$	154.0623	-0.6	13.65	21	C00135
L-Phenylalanine	$C_9H_{11}NO_2$	164.0716	0.6	8.98	28	C00079
trans-Aconitate	$C_6H_6O_6$	173.0092	0.0	0.78	3771	C02341
N2-Acetyl-L-ornithine	$C_7H_{14}N_2O_3$	173.0932	0.0	10.94	3303	C00437
L-Arginine	$C_6H_{14}N_4O_2$	173.1043	0.6	13.89	13	C00062
Citrulline	$C_6H_{13}N_3O_3$	174.0884	0.0	10.96	16	C00327
L-Tyrosine	$C_9H_{11}NO_3$	180.0666	0.0	9.32	34	C00082
Citric acid	$C_6H_8O_7$	191.0207	-5.2	1.11	124	C00158
L-Tryptophan	$C_{11}H_{12}N_2O_2$	203.0824	1.0	9.35	33	C00078
Glycerylphosphorylethanolamine	$C_5H_{14}NO_6P$	214.0486	0.0	8.47	5151	C01233
Nicotinamide adenine dinucleotide (NAD)	$C_{21}H_{28}N_7O_{14}P_2$	663.1045	7.8	10.94	101	C00003
